# 4,4′-[4,4′-(Perfluoro­propane-2,2-di­yl)bis­(4,1-phenyl­eneoxy)]dianiline

**DOI:** 10.1107/S1600536809020121

**Published:** 2009-06-06

**Authors:** Haq Nawaz, Zareen Akhter, Michael Bolte, Humaira M. Siddiqui

**Affiliations:** a Department of Chemistry, Quaid-I-Azam University, Islamabad 45320, Pakistan; b Institut für Anorganische Chemie, J. W. Goethe-Universität Frankfurt, Max-von-Laue-Strasse 7, 60438 Frankfurt/Main, Germany

## Abstract

In the title compound, C_27_H_20_F_6_N_2_O_2_, the dihedral angles between the planes of the aromatic rings connected by the ether O atoms are 84.13 (8) and 75.06 (9)°. The crystal structure is stabilized by N—H⋯O and N—H⋯F hydrogen bonds.

## Related literature

For background to the properties and applications of polyimides, see: Jiang *et al.* (2008 ▶); Matsuura *et al.* (1991 ▶); Nakamura *et al.* (2001 ▶); Stoessel *et al.* (1998 ▶); Zhao *et al.* (2008 ▶). For related structures, see: Nawaz *et al.* (2008 ▶); Bocelli & Cantoni (1989 ▶).
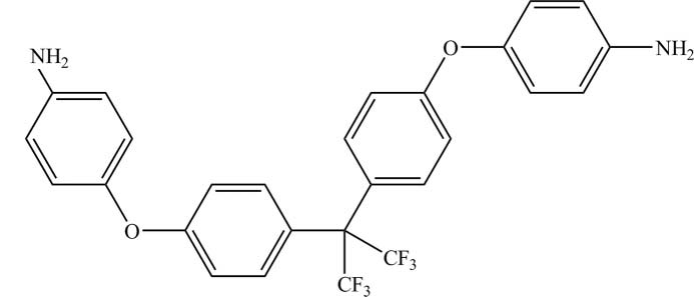


## Experimental

### 

#### Crystal data

C_27_H_20_F_6_N_2_O_2_
*M* *_r_* = 518.45
Orthorhombic, 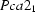

*a* = 11.6914 (12) Å
*b* = 25.641 (2) Å
*c* = 7.7625 (7) Å
*V* = 2327.0 (4) Å^3^
*Z* = 4
Mo *K*α radiation
μ = 0.13 mm^−1^
*T* = 173 K
0.28 × 0.13 × 0.08 mm

#### Data collection

Stoe IPDSII two-circle diffractometer
Absorption correction: none
8855 measured reflections
2875 independent reflections
2186 reflections with *I* > 2σ(*I*)
*R*_int_ = 0.053

#### Refinement

*R*[*F* ^2^ > 2σ(*F* ^2^)] = 0.037
*wR*(*F* ^2^) = 0.088
*S* = 0.92
2875 reflections
351 parameters
1 restraint
H atoms treated by a mixture of independent and constrained refinement
Δρ_max_ = 0.20 e Å^−3^
Δρ_min_ = −0.18 e Å^−3^

### 

Data collection: *X-AREA* (Stoe & Cie, 2001 ▶); cell refinement: *X-AREA*; data reduction: *X-AREA*; program(s) used to solve structure: *SHELXS97* (Sheldrick, 2008 ▶); program(s) used to refine structure: *SHELXL97* (Sheldrick, 2008 ▶); molecular graphics: *XP* in *SHELXTL-Plus* (Sheldrick, 2008 ▶); software used to prepare material for publication: *SHELXL97*.

## Supplementary Material

Crystal structure: contains datablocks I, global. DOI: 10.1107/S1600536809020121/pv2158sup1.cif

Structure factors: contains datablocks I. DOI: 10.1107/S1600536809020121/pv2158Isup2.hkl

Additional supplementary materials:  crystallographic information; 3D view; checkCIF report

## Figures and Tables

**Table 1:** Hydrogen-bond geometry (Å, °)

*D*—H⋯*A*	*D*—H	H⋯*A*	*D*⋯*A*	*D*—H⋯*A*
N1—H1*A*⋯F1^i^	1.03 (5)	2.41 (5)	3.404 (4)	164 (4)
N1—H1*B*⋯O1^ii^	0.92 (3)	2.24 (4)	3.083 (3)	151 (3)
N2—H2*B*⋯F2^iii^	0.86 (9)	3.12 (8)	3.462 (4)	106 (7)

Symmetry codes: (i) 
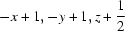
; (ii) 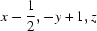; (iii) 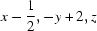.

**Fig. 1.**
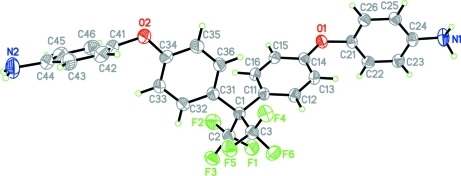
Perspective view of the title compound with the atom numbering; displacement ellipsoids are drawn at the 50% probability level.

## supplementary crystallographic information

### Comment

Polyimides are well known for their excellent thermal and oxidative stability as well as their excellent mechanical properties (Stoessel *et al.*, 1998) suited for use as matrix resins, adhesives and coatings for high-performance applications in the aerospace and electronics industries (Nakamura *et al.*, 2001). These advantages simultaneously give rise to low solubility and poor processability, which can be overcome by incorporation of new functional groups (Matsuura *et al.*, 1991). Many chemists have introduced CF_3_ in polyimides backbone either by means of diamine or dianhydride unit to overcome the solubility issues (Zhao *et al.*, 2008; Jiang *et al.*, 2008). Continuing our investigations in this important area  (Nawaz *et al.*, 2008), we have prepared the title compound, (I), which is also a monomer diamine containing two CF_3_ groups incorporated to enhance the solubility of the resulting polyimides.

The structure of the title compound is presented in Fig. 1. Its bond lenghts and bond angles agree with the corresponding bond lengths and bond angles reported for closely related structures (Nawaz *et al.*, 2008; Bocelli & Cantoni, 1989. The crystal structure of the title compound is stabilized by N—H···O and N—H···F hydrogen bonds; details have been provided in Table 1.

### Experimental

4,4'-(Perfluoropropane-2,2-diyl)bis((4-nitrophenoxy)benzene) (2.00 g, 3.98 mmol) was reduced to corresponding diamine using 10 mL hydrazine and 0.10 g Pd—C as catalyst in 80 mL ethanol under reflux for 24 h. The reaction mixture was filtered and solvent was evaporated to obtain the crude product. It was later recrystallized from absolute ethanol. (Yield 1.56 g; 76%, m.p = 428 (2) K).

### Refinement

In the absence of anomalous scatterers, Friedel pairs (1848) were merged prior to refinement. All H atoms could be located by difference Fourier synthesis. Those bonded to C were refined with fixed individual displacement parameters [U(H) = 1.2 *U*_eq_(C)] using a riding model with C—H = 0.95 Å. The H atoms bonded to N were refined isotropically.

### Crystal data

**Table:**

C_27_H_20_F_6_N_2_O_2_	*D*_x_ = 1.480 Mg m^−^^3^
*M**_r_* = 518.45	Melting point: 428(2) K
Orthorhombic, *P**c**a*2_1_	Mo *K*α radiation, λ = 0.71073 Å
Hall symbol: P 2c -2ac	Cell parameters from 6984 reflections
*a* = 11.6914 (12) Å	θ = 2.4–27.8°
*b* = 25.641 (2) Å	µ = 0.13 mm^−^^1^
*c* = 7.7625 (7) Å	*T* = 173 K
*V* = 2327.0 (4) Å^3^	Plate, colourless
*Z* = 4	0.28 × 0.13 × 0.08 mm
*F*(000) = 1064	

### Data collection

**Table:**

Stoe IPDSII two-circle diffractometer	2186 reflections with *I* > 2σ(*I*)
Radiation source: fine-focus sealed tube	*R*_int_ = 0.053
graphite	θ_max_ = 27.6°, θ_min_ = 3.0°
ω scans	*h* = −14→15
8855 measured reflections	*k* = −31→33
2875 independent reflections	*l* = −7→10

### Refinement

**Table:**

Refinement on *F*^2^	Secondary atom site location: difference Fourier map
Least-squares matrix: full	Hydrogen site location: inferred from neighbouring sites
*R*[*F*^2^ > 2σ(*F*^2^)] = 0.037	H atoms treated by a mixture of independent and constrained refinement
*wR*(*F*^2^) = 0.088	*w* = 1/[σ^2^(*F*_o_^2^) + (0.0536*P*)^2^] where *P* = (*F*_o_^2^ + 2*F*_c_^2^)/3
*S* = 0.92	(Δ/σ)_max_ < 0.001
2875 reflections	Δρ_max_ = 0.20 e Å^−^^3^
351 parameters	Δρ_min_ = −0.17 e Å^−^^3^
1 restraint	Extinction correction: *SHELXL97* (Sheldrick, 2008), Fc^*^=kFc[1+0.001xFc^2^λ^3^/sin(2θ)]^-1/4^
Primary atom site location: structure-invariant direct methods	Extinction coefficient: 0.0185 (15)

### Special details

**Table:**

Geometry. All e.s.d.'s (except the e.s.d. in the dihedral angle between two l.s. planes) are estimated using the full covariance matrix. The cell e.s.d.'s are taken into account individually in the estimation of e.s.d.'s in distances, angles and torsion angles; correlations between e.s.d.'s in cell parameters are only used when they are defined by crystal symmetry. An approximate (isotropic) treatment of cell e.s.d.'s is used for estimating e.s.d.'s involving l.s. planes.
Refinement. Refinement of *F*^2^ against ALL reflections. The weighted *R*-factor *wR* and goodness of fit *S* are based on *F*^2^, conventional *R*-factors *R* are based on *F*, with *F* set to zero for negative *F*^2^. The threshold expression of *F*^2^ &gt; σ(*F*^2^) is used only for calculating *R*-factors(gt) *etc*. and is not relevant to the choice of reflections for refinement. *R*-factors based on *F*^2^ are statistically about twice as large as those based on *F*, and *R*- factors based on ALL data will be even larger.

### Fractional atomic coordinates and isotropic or equivalent isotropic displacement parameters (Å^2^)

**Table:**

	*x*	*y*	*z*	*U*_iso_*/*U*_eq_	
O1	0.51023 (15)	0.60137 (7)	0.7563 (3)	0.0351 (4)	
O2	0.3320 (2)	0.94336 (7)	0.4375 (3)	0.0535 (6)	
N1	0.2707 (2)	0.41299 (10)	0.7968 (4)	0.0497 (7)	
H1A	0.294 (4)	0.3848 (16)	0.709 (8)	0.092 (14)*	
H1B	0.194 (3)	0.4165 (13)	0.818 (5)	0.055 (10)*	
N2	0.3956 (4)	1.14019 (12)	0.1429 (6)	0.0689 (10)	
H2A	0.476 (5)	1.151 (2)	0.154 (9)	0.112 (19)*	
H2B	0.351 (7)	1.149 (3)	0.059 (14)	0.19 (4)*	
C1	0.4885 (2)	0.74565 (9)	0.1928 (4)	0.0310 (5)	
C2	0.6084 (2)	0.74418 (10)	0.1076 (4)	0.0362 (6)	
C3	0.3992 (2)	0.73222 (11)	0.0522 (4)	0.0359 (6)	
F1	0.64236 (14)	0.69487 (6)	0.0771 (3)	0.0469 (4)	
F2	0.68741 (13)	0.76594 (7)	0.2095 (3)	0.0481 (4)	
F3	0.61416 (15)	0.76919 (6)	−0.0438 (2)	0.0451 (4)	
F4	0.29427 (12)	0.72737 (6)	0.1216 (2)	0.0419 (4)	
F5	0.39137 (15)	0.76959 (6)	−0.0682 (2)	0.0456 (4)	
F6	0.42137 (14)	0.68753 (6)	−0.0311 (2)	0.0443 (4)	
C11	0.4892 (2)	0.70497 (9)	0.3394 (3)	0.0300 (5)	
C12	0.4286 (2)	0.65785 (10)	0.3360 (4)	0.0344 (6)	
H12	0.3832	0.6497	0.2382	0.041*	
C13	0.4334 (2)	0.62297 (10)	0.4718 (4)	0.0338 (6)	
H13	0.3908	0.5914	0.4670	0.041*	
C14	0.5001 (2)	0.63392 (10)	0.6149 (4)	0.0312 (5)	
C15	0.5622 (2)	0.68025 (10)	0.6214 (4)	0.0337 (5)	
H15	0.6091	0.6878	0.7182	0.040*	
C16	0.5553 (2)	0.71515 (10)	0.4866 (4)	0.0333 (5)	
H16	0.5965	0.7470	0.4935	0.040*	
C21	0.4462 (2)	0.55454 (9)	0.7554 (4)	0.0324 (5)	
C22	0.4912 (2)	0.51051 (10)	0.6797 (4)	0.0350 (6)	
H22	0.5622	0.5120	0.6205	0.042*	
C23	0.4310 (2)	0.46357 (10)	0.6910 (4)	0.0368 (6)	
H23	0.4615	0.4331	0.6389	0.044*	
C24	0.3270 (2)	0.46080 (10)	0.7774 (4)	0.0353 (6)	
C25	0.2831 (2)	0.50635 (10)	0.8518 (4)	0.0352 (6)	
H25	0.2119	0.5052	0.9104	0.042*	
C26	0.3422 (2)	0.55313 (10)	0.8411 (4)	0.0346 (6)	
H26	0.3118	0.5839	0.8919	0.042*	
C31	0.4565 (2)	0.80045 (10)	0.2600 (4)	0.0328 (5)	
C32	0.4944 (2)	0.84636 (10)	0.1832 (4)	0.0398 (6)	
H32	0.5476	0.8446	0.0908	0.048*	
C33	0.4555 (3)	0.89490 (10)	0.2398 (4)	0.0433 (7)	
H33	0.4831	0.9260	0.1876	0.052*	
C34	0.3765 (3)	0.89754 (11)	0.3727 (4)	0.0400 (6)	
C35	0.3361 (2)	0.85234 (11)	0.4487 (4)	0.0424 (7)	
H35	0.2808	0.8541	0.5381	0.051*	
C36	0.3769 (2)	0.80464 (11)	0.3932 (4)	0.0372 (6)	
H36	0.3499	0.7738	0.4473	0.045*	
C41	0.3554 (3)	0.99075 (10)	0.3522 (4)	0.0415 (7)	
C42	0.2705 (3)	1.01213 (11)	0.2503 (4)	0.0463 (7)	
H42	0.2021	0.9932	0.2290	0.056*	
C43	0.2852 (3)	1.06081 (11)	0.1800 (5)	0.0475 (7)	
H43	0.2264	1.0754	0.1107	0.057*	
C44	0.3851 (3)	1.08902 (11)	0.2087 (5)	0.0465 (7)	
C45	0.4708 (3)	1.06653 (12)	0.3094 (4)	0.0468 (8)	
H45	0.5401	1.0850	0.3287	0.056*	
C46	0.4560 (3)	1.01741 (12)	0.3816 (4)	0.0449 (7)	
H46	0.5145	1.0024	0.4505	0.054*	

### Atomic displacement parameters (Å^2^)

**Table:**

	*U*^11^	*U*^22^	*U*^33^	*U*^12^	*U*^13^	*U*^23^
O1	0.0375 (9)	0.0326 (9)	0.0351 (10)	−0.0058 (7)	−0.0020 (8)	0.0054 (8)
O2	0.0792 (15)	0.0282 (10)	0.0530 (14)	0.0055 (10)	0.0246 (12)	0.0000 (10)
N1	0.0423 (14)	0.0346 (12)	0.072 (2)	−0.0096 (11)	0.0017 (13)	0.0036 (13)
N2	0.086 (2)	0.0347 (14)	0.086 (3)	0.0048 (15)	0.023 (2)	0.0115 (15)
C1	0.0305 (12)	0.0283 (12)	0.0343 (13)	0.0002 (9)	0.0009 (11)	−0.0001 (12)
C2	0.0378 (13)	0.0338 (13)	0.0371 (14)	0.0017 (11)	0.0025 (12)	0.0031 (13)
C3	0.0374 (13)	0.0331 (14)	0.0373 (14)	0.0044 (11)	−0.0001 (12)	0.0043 (12)
F1	0.0455 (9)	0.0371 (8)	0.0581 (11)	0.0115 (7)	0.0114 (8)	0.0019 (8)
F2	0.0327 (8)	0.0585 (11)	0.0532 (10)	−0.0074 (7)	0.0024 (8)	0.0000 (9)
F3	0.0476 (9)	0.0453 (9)	0.0424 (9)	0.0053 (7)	0.0136 (8)	0.0067 (8)
F4	0.0317 (7)	0.0452 (9)	0.0489 (10)	0.0001 (6)	−0.0052 (8)	0.0057 (8)
F5	0.0510 (10)	0.0442 (9)	0.0416 (10)	0.0014 (7)	−0.0083 (8)	0.0118 (8)
F6	0.0540 (10)	0.0379 (8)	0.0409 (9)	0.0024 (7)	−0.0053 (8)	−0.0070 (8)
C11	0.0305 (11)	0.0263 (12)	0.0333 (14)	0.0002 (9)	0.0006 (11)	0.0016 (11)
C12	0.0351 (12)	0.0307 (13)	0.0374 (15)	−0.0018 (10)	−0.0062 (11)	−0.0007 (12)
C13	0.0337 (13)	0.0286 (12)	0.0392 (15)	−0.0045 (11)	−0.0032 (12)	0.0020 (12)
C14	0.0305 (11)	0.0292 (12)	0.0339 (13)	0.0020 (10)	0.0017 (11)	0.0030 (11)
C15	0.0344 (12)	0.0325 (13)	0.0342 (13)	−0.0036 (10)	−0.0009 (12)	−0.0026 (11)
C16	0.0359 (13)	0.0277 (11)	0.0364 (14)	−0.0029 (10)	−0.0019 (12)	−0.0021 (11)
C21	0.0324 (12)	0.0298 (12)	0.0349 (14)	−0.0020 (10)	−0.0028 (11)	0.0051 (11)
C22	0.0338 (12)	0.0374 (14)	0.0339 (14)	0.0034 (10)	0.0034 (11)	0.0018 (12)
C23	0.0385 (13)	0.0310 (12)	0.0410 (15)	0.0042 (11)	−0.0003 (12)	0.0007 (13)
C24	0.0331 (12)	0.0325 (13)	0.0404 (15)	−0.0033 (11)	−0.0067 (12)	0.0045 (12)
C25	0.0304 (12)	0.0370 (13)	0.0383 (15)	0.0001 (10)	0.0031 (12)	0.0028 (12)
C26	0.0348 (12)	0.0323 (13)	0.0368 (15)	0.0029 (11)	0.0025 (11)	0.0028 (12)
C31	0.0324 (12)	0.0303 (12)	0.0357 (14)	0.0001 (10)	0.0019 (11)	0.0026 (12)
C32	0.0472 (14)	0.0330 (13)	0.0393 (15)	−0.0003 (11)	0.0110 (13)	0.0012 (13)
C33	0.0589 (17)	0.0275 (13)	0.0435 (17)	−0.0018 (12)	0.0133 (15)	0.0013 (12)
C34	0.0516 (16)	0.0305 (13)	0.0379 (15)	0.0050 (12)	0.0073 (14)	−0.0039 (12)
C35	0.0427 (15)	0.0374 (15)	0.0472 (17)	0.0011 (12)	0.0141 (14)	0.0002 (14)
C36	0.0361 (13)	0.0310 (13)	0.0446 (16)	0.0005 (11)	0.0089 (12)	0.0022 (12)
C41	0.0557 (16)	0.0294 (13)	0.0393 (16)	0.0005 (12)	0.0093 (14)	−0.0019 (13)
C42	0.0504 (17)	0.0419 (15)	0.0466 (18)	−0.0015 (13)	−0.0028 (15)	−0.0074 (14)
C43	0.0514 (17)	0.0450 (16)	0.0460 (18)	0.0069 (13)	−0.0021 (15)	0.0023 (15)
C44	0.0630 (18)	0.0306 (13)	0.0460 (17)	0.0040 (13)	0.0134 (16)	−0.0006 (14)
C45	0.0468 (16)	0.0398 (15)	0.054 (2)	−0.0033 (13)	0.0049 (14)	−0.0118 (14)
C46	0.0455 (15)	0.0452 (16)	0.0438 (17)	0.0068 (13)	0.0022 (14)	−0.0042 (14)

### Geometric parameters (Å, °)

**Table:**

O1—C14	1.384 (3)	C21—C22	1.377 (4)
O1—C21	1.415 (3)	C21—C26	1.386 (4)
O2—C34	1.380 (3)	C22—C23	1.397 (4)
O2—C41	1.411 (3)	C22—H22	0.9500
N1—C24	1.400 (3)	C23—C24	1.390 (4)
N1—H1A	1.03 (5)	C23—H23	0.9500
N1—H1B	0.92 (3)	C24—C25	1.400 (4)
N2—C44	1.413 (4)	C25—C26	1.387 (4)
N2—H2A	0.99 (6)	C25—H25	0.9500
N2—H2B	0.86 (9)	C26—H26	0.9500
C1—C11	1.544 (3)	C31—C32	1.392 (4)
C1—C31	1.545 (3)	C31—C36	1.396 (4)
C1—C3	1.548 (4)	C32—C33	1.396 (4)
C1—C2	1.551 (3)	C32—H32	0.9500
C2—F2	1.338 (3)	C33—C34	1.386 (4)
C2—F3	1.340 (3)	C33—H33	0.9500
C2—F1	1.346 (3)	C34—C35	1.384 (4)
C3—F6	1.341 (3)	C35—C36	1.382 (4)
C3—F5	1.342 (3)	C35—H35	0.9500
C3—F4	1.346 (3)	C36—H36	0.9500
C11—C12	1.401 (3)	C41—C46	1.379 (4)
C11—C16	1.404 (4)	C41—C42	1.383 (5)
C12—C13	1.384 (4)	C42—C43	1.373 (4)
C12—H12	0.9500	C42—H42	0.9500
C13—C14	1.386 (4)	C43—C44	1.391 (4)
C13—H13	0.9500	C43—H43	0.9500
C14—C15	1.393 (3)	C44—C45	1.396 (5)
C15—C16	1.379 (4)	C45—C46	1.390 (4)
C15—H15	0.9500	C45—H45	0.9500
C16—H16	0.9500	C46—H46	0.9500
			
C14—O1—C21	117.6 (2)	C23—C22—H22	120.4
C34—O2—C41	119.3 (2)	C24—C23—C22	121.0 (2)
C24—N1—H1A	115 (3)	C24—C23—H23	119.5
C24—N1—H1B	113 (2)	C22—C23—H23	119.5
H1A—N1—H1B	116 (3)	C23—C24—N1	120.5 (3)
C44—N2—H2A	108 (3)	C23—C24—C25	118.5 (2)
C44—N2—H2B	118 (5)	N1—C24—C25	120.9 (3)
H2A—N2—H2B	124 (6)	C26—C25—C24	121.0 (2)
C11—C1—C31	111.5 (2)	C26—C25—H25	119.5
C11—C1—C3	111.9 (2)	C24—C25—H25	119.5
C31—C1—C3	106.1 (2)	C21—C26—C25	119.2 (2)
C11—C1—C2	107.1 (2)	C21—C26—H26	120.4
C31—C1—C2	112.6 (2)	C25—C26—H26	120.4
C3—C1—C2	107.7 (2)	C32—C31—C36	117.7 (2)
F2—C2—F3	106.5 (2)	C32—C31—C1	123.2 (2)
F2—C2—F1	107.0 (2)	C36—C31—C1	118.8 (2)
F3—C2—F1	106.3 (2)	C31—C32—C33	121.0 (3)
F2—C2—C1	111.2 (2)	C31—C32—H32	119.5
F3—C2—C1	114.0 (2)	C33—C32—H32	119.5
F1—C2—C1	111.4 (2)	C34—C33—C32	119.7 (3)
F6—C3—F5	106.7 (2)	C34—C33—H33	120.2
F6—C3—F4	106.9 (2)	C32—C33—H33	120.2
F5—C3—F4	106.4 (2)	O2—C34—C35	115.4 (3)
F6—C3—C1	113.6 (2)	O2—C34—C33	124.3 (3)
F5—C3—C1	112.2 (2)	C35—C34—C33	120.2 (3)
F4—C3—C1	110.7 (2)	C36—C35—C34	119.4 (3)
C12—C11—C16	117.0 (2)	C36—C35—H35	120.3
C12—C11—C1	124.5 (2)	C34—C35—H35	120.3
C16—C11—C1	118.5 (2)	C35—C36—C31	122.0 (3)
C13—C12—C11	121.5 (2)	C35—C36—H36	119.0
C13—C12—H12	119.3	C31—C36—H36	119.0
C11—C12—H12	119.3	C46—C41—C42	120.7 (3)
C12—C13—C14	120.2 (2)	C46—C41—O2	121.0 (3)
C12—C13—H13	119.9	C42—C41—O2	118.1 (3)
C14—C13—H13	119.9	C43—C42—C41	119.9 (3)
O1—C14—C13	124.2 (2)	C43—C42—H42	120.1
O1—C14—C15	116.2 (2)	C41—C42—H42	120.1
C13—C14—C15	119.7 (2)	C42—C43—C44	120.9 (3)
C16—C15—C14	119.7 (3)	C42—C43—H43	119.5
C16—C15—H15	120.2	C44—C43—H43	119.5
C14—C15—H15	120.2	C43—C44—C45	118.5 (3)
C15—C16—C11	122.0 (2)	C43—C44—N2	119.8 (3)
C15—C16—H16	119.0	C45—C44—N2	121.6 (3)
C11—C16—H16	119.0	C46—C45—C44	120.7 (3)
C22—C21—C26	121.2 (2)	C46—C45—H45	119.6
C22—C21—O1	119.7 (2)	C44—C45—H45	119.6
C26—C21—O1	118.9 (2)	C41—C46—C45	119.3 (3)
C21—C22—C23	119.1 (2)	C41—C46—H46	120.4
C21—C22—H22	120.4	C45—C46—H46	120.4
			
C11—C1—C2—F2	−72.8 (3)	O1—C21—C22—C23	175.2 (3)
C31—C1—C2—F2	50.2 (3)	C21—C22—C23—C24	−0.1 (4)
C3—C1—C2—F2	166.8 (2)	C22—C23—C24—N1	−176.2 (3)
C11—C1—C2—F3	166.8 (2)	C22—C23—C24—C25	0.6 (4)
C31—C1—C2—F3	−70.3 (3)	C23—C24—C25—C26	−0.6 (4)
C3—C1—C2—F3	46.3 (3)	N1—C24—C25—C26	176.2 (3)
C11—C1—C2—F1	46.5 (3)	C22—C21—C26—C25	0.6 (4)
C31—C1—C2—F1	169.4 (2)	O1—C21—C26—C25	−175.2 (3)
C3—C1—C2—F1	−74.0 (3)	C24—C25—C26—C21	0.0 (4)
C11—C1—C3—F6	−64.1 (3)	C11—C1—C31—C32	151.6 (3)
C31—C1—C3—F6	174.1 (2)	C3—C1—C31—C32	−86.4 (3)
C2—C1—C3—F6	53.3 (3)	C2—C1—C31—C32	31.2 (4)
C11—C1—C3—F5	174.8 (2)	C11—C1—C31—C36	−35.1 (3)
C31—C1—C3—F5	52.9 (3)	C3—C1—C31—C36	87.0 (3)
C2—C1—C3—F5	−67.9 (3)	C2—C1—C31—C36	−155.4 (3)
C11—C1—C3—F4	56.1 (3)	C36—C31—C32—C33	1.1 (4)
C31—C1—C3—F4	−65.7 (3)	C1—C31—C32—C33	174.6 (3)
C2—C1—C3—F4	173.5 (2)	C31—C32—C33—C34	−1.1 (5)
C31—C1—C11—C12	127.0 (3)	C41—O2—C34—C35	−171.0 (3)
C3—C1—C11—C12	8.3 (3)	C41—O2—C34—C33	8.4 (5)
C2—C1—C11—C12	−109.4 (3)	C32—C33—C34—O2	−179.5 (3)
C31—C1—C11—C16	−53.5 (3)	C32—C33—C34—C35	−0.2 (5)
C3—C1—C11—C16	−172.2 (2)	O2—C34—C35—C36	−179.3 (3)
C2—C1—C11—C16	70.1 (3)	C33—C34—C35—C36	1.3 (5)
C16—C11—C12—C13	0.2 (4)	C34—C35—C36—C31	−1.2 (5)
C1—C11—C12—C13	179.7 (2)	C32—C31—C36—C35	0.0 (4)
C11—C12—C13—C14	−0.7 (4)	C1—C31—C36—C35	−173.7 (3)
C21—O1—C14—C13	−1.5 (4)	C34—O2—C41—C46	−83.0 (4)
C21—O1—C14—C15	178.6 (2)	C34—O2—C41—C42	102.7 (3)
C12—C13—C14—O1	−179.9 (2)	C46—C41—C42—C43	−1.2 (5)
C12—C13—C14—C15	0.1 (4)	O2—C41—C42—C43	173.1 (3)
O1—C14—C15—C16	−179.0 (2)	C41—C42—C43—C44	0.4 (5)
C13—C14—C15—C16	1.0 (4)	C42—C43—C44—C45	0.7 (5)
C14—C15—C16—C11	−1.6 (4)	C42—C43—C44—N2	−176.5 (3)
C12—C11—C16—C15	0.9 (4)	C43—C44—C45—C46	−1.0 (5)
C1—C11—C16—C15	−178.6 (2)	N2—C44—C45—C46	176.1 (3)
C14—O1—C21—C22	87.0 (3)	C42—C41—C46—C45	0.8 (5)
C14—O1—C21—C26	−97.2 (3)	O2—C41—C46—C45	−173.3 (3)
C26—C21—C22—C23	−0.5 (4)	C44—C45—C46—C41	0.3 (5)

### Hydrogen-bond geometry (Å, °)

**Table:**

*D*—H···*A*	*D*—H	H···*A*	*D*···*A*	*D*—H···*A*
N1—H1A···F1^i^	1.03 (5)	2.41 (5)	3.404 (4)	164 (4)
N1—H1B···O1^ii^	0.92 (3)	2.24 (4)	3.083 (3)	151 (3)
N2—H2B···F2^iii^	0.86 (9)	3.12 (8)	3.462 (4)	106 (7)

Symmetry codes: (i) −*x*+1, −*y*+1, *z*+1/2; (ii) *x*−1/2, −*y*+1, *z*; (iii) *x*−1/2, −*y*+2, *z*.
